# Lymphocyte‐C‐Reactive Protein Ratio as Promising New Marker for Predicting Surgical Site Infection in Children With Ulcerative Colitis

**DOI:** 10.1002/ags3.70236

**Published:** 2026-05-25

**Authors:** Yuhki Koike, Koki Higashi, Yuki Sato, Yuka Nagano, Tadanobu Shimura, Takahito Kitajima, Kohei Matsushita, Yoshiki Okita, Yoshinaga Okugawa, Yuji Toiyama

**Affiliations:** ^1^ Department of Gastrointestinal and Pediatric Surgery Mie University Graduate School of Medicine Tsu Mie Japan; ^2^ Department of Genomic Medicine Mie University Graduate School of Medicine Tsu Mie Japan

**Keywords:** inflammatory bowel disease, lymphocyte C‐reactive protein ratio, pediatric surgery, surgical site infection, ulcerative colitis

## Abstract

**Background:**

Surgical site infection (SSI) is a major complication after ileal pouch–anal anastomosis (IPAA) in pediatric ulcerative colitis (UC), significantly impairing quality of life. The lymphocyte‐to–C‐reactive protein ratio (LCR), a composite marker of systemic inflammation and immune/nutritional status, has emerged as a potential predictor of postoperative outcomes. This study assessed the utility of preoperative LCR in predicting SSI in pediatric UC.

**Methods:**

We retrospectively reviewed pediatric UC patients who underwent IPAA at Mie University Hospital between 2000 and 2024. Preoperative LCR values were analyzed, and the optimal cutoff for SSI prediction was determined using receiver operating characteristic (ROC) analysis. Multivariate logistic regression was performed to identify independent risk factors. SSI was defined according to CDC criteria within 30 days postoperatively.

**Results:**

Among 57 patients, 11 (19.3%) developed SSI. The incidence was higher in three‐stage procedures than in two‐stage procedures (22.7% vs. 17.1%). In both groups, preoperative LCR was significantly lower in SSI‐positive patients. ROC analysis demonstrated good discrimination (AUC: 0.80), with an optimal cutoff of LCR < 5000 (sensitivity: 90.9%, specificity: 71.7%). Multivariate analysis confirmed LCR < 5000 as an independent predictor of SSI (odds ratio [OR]: 6.34, 95% CI: 1.23–48.2, *p* = 0.027).

**Conclusion:**

Preoperative LCR is a simple, objective biomarker that reliably predicts SSI risk in pediatric UC patients undergoing IPAA. Incorporating LCR into preoperative risk stratification may enable personalized interventions, including nutritional optimization and tailored prophylaxis, to improve surgical outcomes.

## Graphical abstract

This study identifies the preoperative lymphocyte‐to–C‐reactive protein ratio (LCR) as a potent and independent biomarker for predicting surgical site infection (SSI) in pediatric patients undergoing major surgery for ulcerative colitis. ROC analysis established an optimal cutoff of 5000 (AUC: 0.80), which demonstrated robust predictive performance independent of surgical staging and cumulative preoperative steroid exposure. These results suggest that incorporating LCR into routine clinical practice enables personalized risk stratification and targeted interventions to improve surgical outcomes in children.

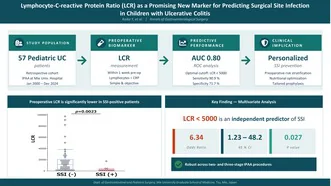

## 1 Introduction

Ulcerative colitis (UC) is the most common form of inflammatory bowel disease (IBD), characterized by chronic mucosal inflammation of the colon and rectum. Approximately 20% of UC cases are diagnosed during childhood, and pediatric patients typically present with more severe and extensive disease [1]. Consequently, nearly 40% of children with UC ultimately require total proctocolectomy within 10 years of diagnosis [2]. Restorative proctocolectomy with ileal pouch–anal anastomosis (IPAA) has become the standard surgical approach in this setting [3]. Although IPAA is effective, it is associated with a range of postoperative complications, among which surgical site infection (SSI) is one of the most frequent septic events. A retrospective analysis of 136 pediatric patients with UC in Japan who underwent surgery between 2000 and 2012 reported that SSI occurred in 36/136 patients (26%). In this cohort, the SSI incidence varied significantly based on the staged procedure: 19% in two‐stage surgery, and 48% in three‐stage surgery [4].

SSI, particularly organ/space infections such as pelvic sepsis, is strongly linked to pouch failure and contributes to substantial morbidity, prolonged hospitalization, and increased healthcare costs [5]. Established independent risk factors for SSI in UC include high cumulative preoperative steroid exposure (≥ 10 000 mg prednisolone equivalents) and poor general physical status (e.g., American Society of Anesthesiologists [ASA] score ≥ 3) [3]. In addition, surgical strategy, such as three‐stage procedures, has been implicated as an independent predictor of SSI in children. Nevertheless, the identification of simple, objective preoperative biomarkers remains essential for improving risk stratification and optimizing perioperative management [4, 6].

Recently, composite inflammatory markers derived from routine blood counts and acute‐phase reactants have gained attention for their ability to integrate systemic inflammation, nutritional status, and immunosuppression [7]. The lymphocyte‐to–C‐reactive protein ratio (LCR), calculated by dividing the absolute lymphocyte count by the serum C‐reactive protein (CRP) level, represents a simple and objective index [8]. LCR reflects the balance between systemic inflammation (elevated CRP and suppressed lymphocytes) and the patient's nutritional reserve. In adult surgical oncology, preoperative LCR has been validated as an independent predictor of postoperative complications and SSI, particularly in patients undergoing colorectal cancer resection (cutoff ≤ 6000) and esophagogastric junction cancer surgery [8, 9]. However, the predictive utility of LCR has not been established in the pediatric UC population undergoing IPAA. Given its demonstrated prognostic value in other complex abdominal surgeries, we hypothesized that preoperative LCR could serve as a reliable, non‐invasive biomarker for SSI risk in pediatric UC patients. The primary objective of this study was to evaluate the association between preoperative LCR and SSI following total proctocolectomy in children with UC, and to investigate the utility of LCR as a predictive biomarker for postoperative SSI in this population.

## 2 Methods

### 2.1 Study Population

The study cohort comprised 60 consecutive pediatric patients with UC who underwent their initial surgery before the age of 20 at Mie University Hospital, Japan, between January 2000 and December 2024. After exclusion of three patients with incomplete clinical or surgical data, 57 patients were included in the retrospective analysis. The study was approved by the Mie University Hospital Institutional Review Board (Approval No. H2025‐097). Informed consent was obtained via an opt‐out procedure posted on the hospital website; patients who declined were excluded from analysis.

### 2.2 Selection of Staged Surgical Procedure

At our institution, staged operations are selected according to the patient's preoperative condition [10]. The two‐stage approach comprises an initial total proctocolectomy with IPAA and diverting ileostomy, followed by ileostomy closure. The three‐stage approach begins with subtotal colectomy with ileostomy and sigmoid mucous fistula, followed by completion proctectomy with IAA and ileostomy reconstruction, and concludes with ileostomy closure. Choice of two‐ versus three‐stage surgery was based on clinical factors such as massive anal bleeding, toxic megacolon, hemodynamic instability, sepsis, and nutritional status. This means that three‐stage surgery is adopted for serious cases.

### 2.3 Definition of SSI

Medical records were reviewed for superficial incisional, deep incisional, and organ/space SSIs according to Centers for Disease Control and Prevention (CDC) definitions; all three categories were counted as SSI [11]. In accordance with the CDC definitions, every effort was made to accurately identify SSI within 30 days post‐surgery in patients who had been discharged. This process involved a review of hospital records for pediatric patients who were readmitted within this period, with an examination of outpatient surgical records and emergency department reports. In this study, the occurrence of SSI was specifically assessed within 30 days following the first‐stage operation, consistent with the timing of the preoperative LCR measurement.

### 2.4 Clinical Variables and Outcome Measures

Data on patient demographics, potential risk factors, and occurrence of SSI were collected retrospectively. The variables evaluated as potential predictive factors for SSI were gender, onset age of UC, disease duration, age at operation, extent of colitis, preoperative medication usage (total dose of steroids, biologic agents), staged operation (two or three), operation time, operative blood loss, blood transfusion (history of blood transfusion within 1 week before and/or after the date of surgery), ASA classification, preoperative serum albumin level, and LCR value. The preoperative UC activity of the patients was assessed by the Pediatric Ulcerative Colitis Activity Index (PUCAI) [12]. A PUCAI score of 0 was defined as remission, PUCAI 10–30 as mild UC activity, PUCAI 35–60 as moderate UC activity, and PUCAI 65–85 as severe UC activity.

### 2.5 Laboratory Measurements and LCR Calculation

Peripheral blood samples were obtained within 1 week prior to the initial major surgery (total proctocolectomy for the two‐stage group or subtotal colectomy for the three‐stage group). In cases of staged procedures, we utilized the LCR values obtained specifically before the first stage. This approach was adopted because the baseline systemic inflammatory and nutritional status at the initiation of the surgical series is considered to have the most significant impact on initial wound healing and the risk of early septic complications.

The lymphocyte‐to‐CRP ratio (LCR) was calculated as:

$$\mathrm{LCR}=\text{Lymphocyte}\;\text{count}\left(\mathrm{per}\;\mathrm{\mu L}\right)/\text{Serum}\;\mathrm{CRP}\;\text{level}\left(g/\mathrm{dL}\right).$$

### 2.6 Statistical Analysis

Analyses were performed using JMP version 16 (SAS Institute, Cary, NC, USA). Group comparisons used the chi‐square test for categorical variables and the Mann–Whitney *U* test for continuous variables, given non‐normal distributions assessed by *F* tests. Receiver operating characteristic (ROC) curves with Youden's index were used to determine optimal cutoff values for candidate predictors, including LCR. Variables considered in univariate and multivariate logistic regression included sex; age at onset and operation, disease duration, total steroid dose, PUCAI, operation time, and operative blood loss (these items were categorized by median values); extent of colitis; biologic exposure (experienced vs. naive); staged procedure (two vs. three); and perioperative transfusion. Two‐sided *p* values < 0.05 were considered statistically significant. Variables with *p* < 0.05 in univariate analysis were entered into the multivariate model.

## 3 Results

### 3.1 Patient Characteristics

A total of 57 pediatric patients with UC who underwent total proctocolectomy were included in this study. The median age at operation was 14 years (interquartile range [IQR]: 10.5–16 years), and the median preoperative disease duration was 3 years (IQR: 1.3–5 years). The majority of patients had total colitis (53/57). Overall, 11 patients (19.3%) developed SSI postoperatively [Table 1]. Among them, seven were superficial incisional, no deep SSI, and four were organ/space infections. None of these patients developed pelvic sepsis in this study.

**TABLE 1:** Patient characteristics.

Clinical variables	All patients (*n* = 57)
General	
Sex (male/female)	34/24
Age at onset of ulcerative colitis, years (median ± IQR)	11 (7.5–13)
Disease duration, years (median ± IQR)	3 (1.3–5)
Age at operation, years (median ± IQR)	14 (10.5–16)
Extent of colitis (left side/total)	4/53
PUCAI score at surgery (median ± IQR)	35 (20–45)
Total dose of steroids (median ± IQR)	3610 (1900–7500)
Biologic therapy (yes/no)	14/43
Intraoperative	
Type of operation	
Two‐stage	35
Three‐stage	22
Operation time, min (median ± IQR)	284 (234–353)
Operative blood loss, mL (median ± IQR)	180 (49–328)
Blood transfusion (yes/no)	11/46
Postoperative	
Surgical site infection, *n* (%)	11 (19.3%)
Superficial	7
Deep	0
Organ/space	4

Abbreviations: IQR, interquartile range; PUCAI, pediatric ulcerative colitis activity index.

### 3.2 Surgical Procedures and SSI Incidence Rate

**FIGURE 1:**
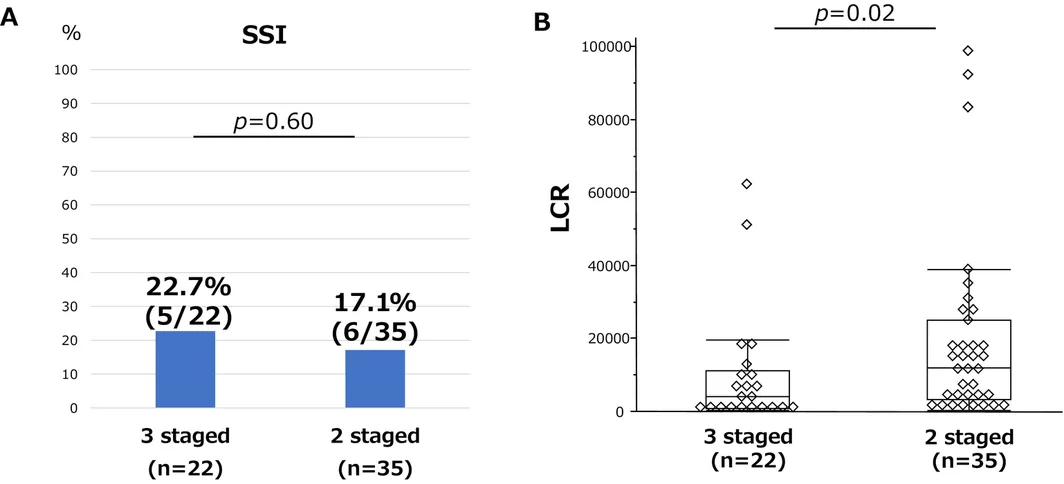
(A) SSI incidence rates in three staged and two staged Surgery. Three‐stage surgery showed a higher incidence of SSI than two‐stage surgery. (B) Comparison of LCR Values in Three‐Stage and Two‐Stage Surgery. In the three‐stage surgery, the LCR value was significantly lower compared to the two‐stage surgery.

The incidence of SSI was compared between three staged and two staged surgical procedures (Figure 1A). Of the total cohort, 35 patients underwent two‐stage surgery, while 22 patients underwent three‐stage surgery. Patients undergoing three‐stage surgery had a higher SSI incidence rate of 22.7% (5/22), compared to 17.1% (6/35) in the two‐stage surgery group. However, no statistically significant difference in SSI incidence rates was observed between the two groups (*p* = 0.60).

### 3.3 Distribution of Preoperative LCR Values by Staging Procedure

Preoperative LCR values were compared between the three‐stage surgery group (*n* = 22) and the two‐stage surgery group (*n* = 35) (Figure 1B). The distribution of LCR values in the three‐stage cohort was significantly lower than that in the two‐stage cohort, reflecting the potentially more complex and severe underlying disease status requiring the staged approach (*p* = 0.02).

### 3.4 Comparison of Clinical and Perioperative Factors Stratified by Surgical Site Infection

The correlation between the presence or absence of SSI and preoperative/intraoperative factors was analyzed (Table 2).

**TABLE 2:** Correlation between clinical variables and SSI in children with ulcerative colitis.

Variables	SSI (+) (*n* = 11)	SSI (−) (*n* = 46)	*p*
Sex			
Male	8	26	0.33
Female	3	20	
Onset ages (years)^a^	9 (1–13)	11 (7.8–13.3)	0.23
Disease duration (years)^a^	3 (2–11)	3 (1.1–5)	0.21
Age at operation (years)^a^	13 (12–14.3)	14 (10–16.3)	0.68
Extent of colitis			
Left side	1	3	0.76
Total	10	43	
PUCAI score at first surgery^a^	40 (15–55)	35 (20–45)	0.78
Total dose of steroids^a^	7500 (1800–9250)	3438 (1950–5275)	0.055
Biologic therapy			
Yes	1	13	0.18
No	10	33	
Two‐staged operation	6	29	0.61
Operation time (min)^a^	253 (202–359)	325 (264–392)	0.15
Operative blood loss (mL)^a^>	165 (92–983)	213 (70–339)	0.73
Three‐staged operation	5	17	
Operation time (min)^a^>	274 (209–295)	257 (212–642)	0.97
Operative blood loss (mL)^a^	56 (23–597)	177 (33–287)	0.94
Blood transfusion			
Yes	5	6	**0.014**
No	6	40	
ASA classification			
1–2	6	39	**0.027**
3–5	5	7	
Preoperative albumin (g/dL)^b^	3.5 (2.8–4.4)	3.6 (3.3–4.2)	0.92
Preoperative LCR^b^	2052 (673–3657)	11 475 (3167–20 928)	**0.0023**

*Note:* Values that met the criterion for statistical significance (*P* < 0.05) are shown in bold.

Abbreviations: LCR, leukocyte C‐reactive protein ratio; PUCAI, pediatric ulcerative colitis activity index.

^a^ IQR: interquartile range.

^b^ The value was within 1 week prior to the initial surgery.

The SSI+ group exhibited a significantly lower median preoperative LCR of 2052 (IQR: 673–3657), compared with 11 475 (IQR: 3167–20 928) in the SSI− group (*p* = 0.0023). This finding indicates that impaired inflammatory and nutritional status, as reflected by a reduced LCR, is associated with an increased risk of SSI. Among intraoperative factors, blood transfusion was significantly correlated with SSI occurrence (*p* = 0.014). Additionally, a higher proportion of patients in the SSI+ group had an ASA classification of ≥ 3 (5/12 [41.6%] vs. 6/45 [13.3%], *p* = 0.027). Other variables did not differ significantly between the two groups in this univariate analysis.

### 3.5 Impact of Surgical Procedure on Preoperative LCR and Risk of SSI

**FIGURE 2:**
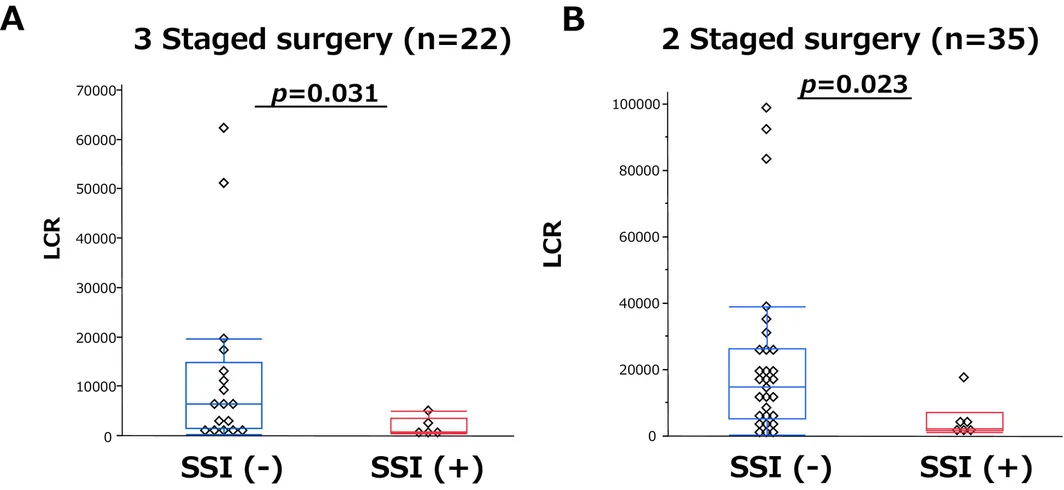
(A) Comparison of LCR values and SSI in three staged operation. LCR values were significantly lower in the SSI (+) group. (B) Comparison of LCR Values and SSI in two staged operation. LCR values were significantly lower in the SSI (+) group.

Because the staging procedure reflects surgical complexity and has been recognized as a factor influencing the risk of postoperative infectious complications in pediatric UC surgery, the predictive value of the LCR for SSI was further evaluated after stratification by operative approach. The cohort was divided into two groups: patients undergoing three‐stage surgery and those undergoing two‐stage surgery. Figure 2A shows the comparison within the three‐stage surgery cohort (*n* = 22). In this subgroup, preoperative LCR values were significantly lower in patients who developed SSI than in those without SSI, with a statistically significant difference (*p* = 0.031). Figure 2B depicts the findings for the two‐stage surgery cohort (*n* = 35). Consistent with the three‐stage group, preoperative LCR values were also significantly lower in SSI+ patients compared with SSI− patients, and this association remained statistically significant (*p* = 0.023). These results indicate that preoperative LCR retains its prognostic utility for predicting SSI risk, regardless of the surgical staging procedure employed in pediatric UC patients.

### 3.6 Predictive Performance of the Preoperative LCR for SSI in Pediatric Ulcerative Colitis Patients

**FIGURE 3:**
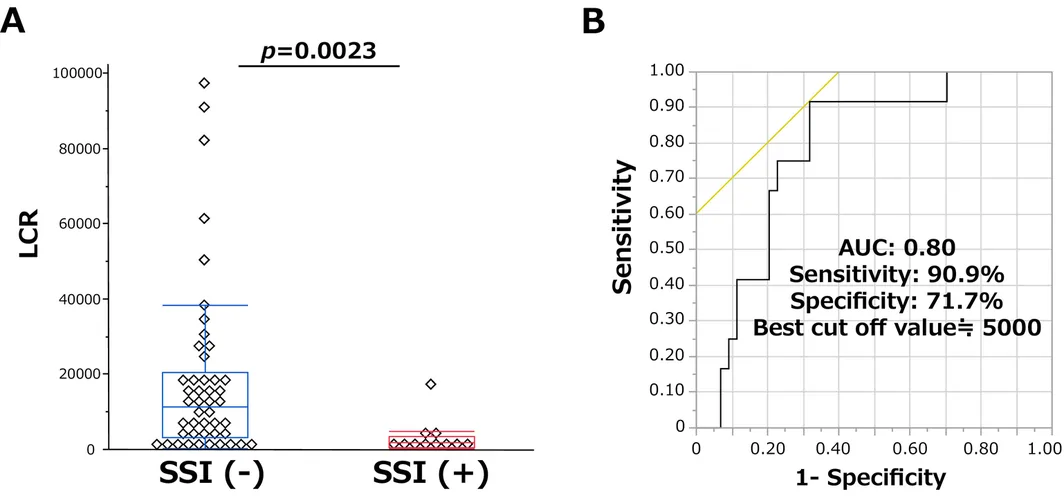
(A) Comparison of LCR values and SSI in children after UC surgery. LCR values were significantly lower in the SSI (+) group. (B) Calculation of the optimal cutoff LCR value for the risk of SSI by ROC analysis. Analysis calculated an LCR value of approximately 5000 as the cut‐off value.

Figure 3A shows the comparison of preoperative LCR values between patients who developed SSI+ and those who did not SSI−. Preoperative LCR values were significantly lower in the SSI+ group compared with the SSI− group (*p* = 0.0023), indicating a strong association between reduced LCR and an increased risk of postoperative infectious complications. These findings suggest that a lower LCR reflects a compromised preoperative inflammatory and nutritional status that predisposes patients to SSI.

### 3.7 Predictive Performance and Optimal Cutoff Value

To evaluate the diagnostic performance of preoperative LCR and to determine an optimal threshold for risk stratification, ROC curve analysis was performed (Figure 3B). The ROC analysis demonstrated good predictive accuracy, with an area under the curve (AUC) of 0.80 for SSI occurrence. The optimal cutoff value of preoperative LCR, defined by maximizing the Youden index, was approximately 5000. Using this threshold (LCR < 5000), the sensitivity for predicting SSI was 90.9%, and the specificity was 71.7%. These results confirm that preoperative LCR is a robust biomarker capable of distinguishing between high‐ and low‐risk pediatric UC patients with respect to postoperative SSI development.

### 3.8 Multivariate Analysis of Risk Factors for SSI

To identify independent preoperative risk factors for SSI, univariate and multivariate logistic regression analyses were performed using categorized variables based on median values, including the LCR threshold of < 5000 established in Figure 3B.

In the univariate analysis (Table 3), a low preoperative LCR (< 5000) emerged as the strongest predictor of SSI ([OR]: 9.30, *p* = 0.0026). Additional factor significantly associated with SSI included an intraoperative blood transfusion (OR: 5.56, *p* = 0.023), and ASA classification ≥ 3 (OR: 4.64, *p* = 0.039). Other variables were not significantly associated with SSI.

**TABLE 3:** Univariate and multivariate analysis of risk factors for surgical site infection.

Variables	Univariate			Multivariate		
	OR	95% CI	*p*	OR	95% CI	*p*
Sex (male)	2.05	0.52–10.3	0.32			
Onset age ≤ 14 years old^a^	2.05	0.51–10.3	0.32			
Ope age ≤ 14 years old^a^	2.44	0.61–12.2	0.21			
Disease duration ≥ 3 years^a^	1.30	0.33–4.93	0.70			
Extent of colitis (total colonic)	1.43	0.08–14.9	0.77			
Total dose of steroids ≥ 3600 mg^a^	3.17	0.80–15.9	0.10			
Biologic therapy (−)	3.94	0.65–75.4	0.15			
PUCAI score at 1st surgery ≥ 35^a^	1.43	0.38–5.60	0.60			
Surgical procedure (three‐staged)	1.42	0.36–5.43	0.61			
Operation time ≥ 286 min	1.91	0.51–8.13	0.34			
Operative blood loss ≥ 180 mL	2.08	0.55–8.88	0.28			
Blood transfusion (+)	5.56	1.27–25.0	**0.023**	2.73	0.44–16.1	0.27
ASA classification ≥ 3	4.64	1.09–20.1	**0.039**	1.69	0.25–9.69	0.57
Preoperative albumin < 3.5^a^ (g/dL)	1.43	0.37–5.60	0.59			
Preoperative LCR < 5000^b^	9.30	2.08–66.2	**0.0026**	6.34	1.23–48.2	**0.027**

*Note:* Values that met the criterion for statistical significance (*P* < 0.05) are shown in bold.

^a^ For the items of onset age, ope age, disease duration, PUCAI score, total dose of steroids, operation time, operative blood loss, albumin, the value were divided by median.

^b^ Regarding LCR, the best cut‐off value in SSI was calculated using ROC analysis.

Multivariate analysis incorporating these variables confirmed that low preoperative LCR (< 5000) was the only independent predictor of SSI (OR: 6.34, 95% CI: 1.23–48.2, *p* = 0.027). Blood transfusion and ASA classification did not retain statistical significance in the multivariate model. These findings underscore the prognostic utility of preoperative LCR, a composite marker of inflammatory and nutritional status, as a non‐invasive predictor of postoperative SSI in pediatric UC patients undergoing total proctocolectomy.

### 3.9 Independence of LCR From Steroid Exposure and Robustness of the Cutoff Value

To address the potential confounding effect of preoperative corticosteroid administration, which may increase the neutrophil percentage and induce relative lymphopenia, we further examined the correlation between the total steroid dose and preoperative LCR values (Figure S1). Analysis revealed no significant correlation between these two variables (*p* = 0.19). Furthermore, to verify the robustness of our findings, we performed a sensitivity analysis using the conventional LCR cutoff value of 6000 validated in adult malignancies [8]. In this revised model, low LCR status again emerged as the sole independent predictor of SSI (Table S1).

## 4 Discussion

The primary finding of the present study is the identification of the LCR as a potent, independent predictive biomarker for SSI following surgery in children with UC. Our results indicate that lower preoperative LCR levels (specifically, an LCR < 5000) are strongly associated with a significantly increased risk of developing SSI.

LCR integrates both inflammatory up‐regulation (reflected by CRP) and potential immune/nutritional down‐regulation (reflected by lymphocyte count) [8, 13]. This dual‐factor approach likely provides a more comprehensive assessment of the patient's systemic physiological stress and immune status compared to individual markers alone. The utility of composite inflammation‐based scores, such as the CRP‐albumin ratio (CAR) and LCR (or CRP‐lymphocyte ratio; CLR), has recently been demonstrated in the context of inflammatory bowel disease (IBD) [7, 13]. Studies in adult patients with acute severe UC (ASUC) receiving infliximab salvage therapy showed that CLR and CAR measured on Day 3 post‐infliximab were superior predictors of colectomy risk compared to conventional scores like the Mayo score, Neutrophil lymphocyte ratio (NLR), and Platelet lymphocyte ratio (PLR) [7]. Furthermore, LCR has been recognized as a reliable predictor for both postoperative complications, including SSI, and long‐term prognosis (Disease‐Free Survival and Overall Survival) in patients undergoing surgery for colorectal cancer (CRC) and esophago‐gastric junction (EGJ) cancer [8, 9]. These findings suggest that LCR is a broadly applicable and objective tool for quantifying systemic risk in surgical oncology and severe inflammation settings.

In the management of pediatric UC, the choice of staged surgery remains a critical decision influencing SSI risk [4]. Our data support the existing evidence that three‐stage procedures are associated with a higher incidence of SSI compared to two‐stage procedures, and three‐stage surgery has previously been identified as an independent predictor of SSI in pediatric UC. Patients requiring three‐stage surgery typically present with more severe conditions, including worse Pediatric Ulcerative Colitis Activity Index (PUCAI) scores, lower preoperative albumin, and higher CRP levels [4]. The finding that low LCR predicts SSI risk across both two‐stage and three‐stage groups suggests that LCR captures the underlying systemic inflammatory and compromised host status regardless of the staging technique used.

The association between preoperative systemic risk factors and SSI in UC is well‐documented, though controversies persist [5, 6]. Our analysis showed LCR < 5000 to be an independent predictor for SSI. This result parallels other key findings in UC surgical risk literature, such as the risk associated with perioperative blood transfusion, which was also a significant risk factor in our univariate analysis. While other studies have identified factors like high ASA physical status (≥ 3) [14] and female sex [4] as SSI predictors in UC populations, LCR serves as a single, objective, and readily available measure integrating the composite risks related to both inflammation and potential malnutrition [15].

Figure 2 demonstrates that LCR levels were significantly lower in the SSI‐positive cohort within both the 2‐stage and 3‐stage subgroups. These results corroborate the validity of LCR as an independent prognostic factor for SSI in pediatric UC, one that remains robust regardless of the operative method. For instance, the 3‐stage procedure is generally associated with a higher risk of SSI based on previous reports. This approach is typically selected for critical cases requiring urgent intervention, such as those with toxic megacolon or uncontrollable hematochezia necessitating blood transfusion. Our results suggest that even among these critically ill patients, a low preoperative LCR indicates a heightened risk of SSI. Consequently, clinical applications for these high‐risk cases might include not only more rigorous postoperative management but also proactive measures such as the escalation of antibiotic regimens or the extension of treatment duration. Conversely, 2‐stage procedures are typically reserved for cases of moderate severity where immediate surgical intervention is not critical, thereby offering a therapeutic window. In such scenarios, a low preoperative LCR could serve as a signal to modify the treatment plan; specifically, clinicians might delay surgery to optimize nutritional status or aggressively control inflammation. In other words, active preoperative interventions aimed at elevating LCR—by enhancing host immunity through nutritional support and reducing inflammatory markers—may potentially mitigate the risk of postoperative SSI. Ultimately, these findings highlight the potential of LCR as a valuable clinical index for guiding personalized therapeutic planning in the surgical management of UC.

The optimal preoperative LCR cutoff identified in this study was 5000, which is slightly lower than the threshold of 6000 validated in adult surgical oncology [8]. This discrepancy likely reflects the unique clinical and physiological context of pediatric UC. First, unlike patients with solid tumors, those with active pediatric UC frequently receive high‐dose corticosteroids, which are known to induce neutrophil‐dominant leukocytosis and relative lymphopenia. This systemic shift in the white blood cell differential likely results in lower baseline LCR values in the UC population compared to oncology cohorts. Importantly, our additional analysis revealed no significant correlation between preoperative LCR values and the total dose of steroid (Figure S1, *p* = 0.19), suggesting that LCR serves as an independent marker of systemic status rather than a mere reflection of steroid exposure. Furthermore, a sensitivity analysis using the conventional cutoff of 6000 confirmed that low LCR status remained the sole independent predictor of SSI in the multivariate model (Table S1). These results underscore the robust predictive performance of LCR in this cohort, regardless of whether a population‐specific or conventional threshold is applied. Consequently, LCR serves as a reliable and versatile biomarker for personalized risk stratification in pediatric patients undergoing major surgery for UC.

We acknowledge that the sample size of 57 patients is a one of the limitations of this study. As a single‐center retrospective study with a relatively small sample size inherent to pediatric research, interpretation requires caution [9, 15] However, total proctocolectomy in pediatric UC is a specialized procedure for a relatively rare population. Given the scarcity of pediatric‐specific data, this cohort represents a clinically significant sample size that allowed for a sufficient analysis of SSI risk. Furthermore, we maintained an exclusive focus on pediatric patients to avoid the confounding effects of adult physiology, as pediatric UC often presents with a more aggressive disease course compared to adults [1, 2]. Future multicenter, prospective studies with larger patient populations are warranted to confirm the generalizability of LCR as a standard SSI predictive biomarker in pediatric UC surgery [15, 16].

In conclusion, the preoperative LCR is a promising, simple, and objective biomarker that independently predicts the risk of SSI in pediatric UC patients undergoing surgery. Incorporating LCR into the preoperative risk assessment algorithm may enhance personalized SSI prevention strategies and ultimately improve postoperative outcomes in this high‐risk population.

## Author Contributions

**Takahito Kitajima:** formal analysis. **Kohei Matsushita:** project administration, data curation. **Yuhki Koike:** writing – review and editing, conceptualization, writing – original draft, project administration, data curation, formal analysis, investigation. **Koki Higashi:** data curation. **Yuki Sato:** data curation. **Yoshinaga Okugawa:** supervision. **Yoshiki Okita:** project administration. **Yuji Toiyama:** conceptualization, writing – review and editing, supervision. **Tadanobu Shimura:** data curation. **Yuka Nagano:** data curation.

## Funding

The authors have nothing to report.

## Ethics Statement

The institutional review board of Mie University Hospital approved the present study (Approval No. H2025‐097).

## Consent

Informed consent was obtained via an opt‐out procedure posted on the hospital website; patients who declined were excluded from analysis.

## Conflicts of Interest

The authors declare no conflicts of interest.

## Supporting information


**Figure S1:** Association between preoperative steroid exposure and LCR. There was no significant correlation between the total dose of preoperative steroids and the LCR values (*p* = 0.19).

**Table S1:** Univariate and multivariate analysis of risk factors for surgical site infection.

## Data Availability

The data that support the findings of this study are available on request from the corresponding author. The data are not publicly available due to privacy or ethical restrictions.
