# Supplementary material for: Lymphocyte‐C‐Reactive Protein Ratio as Promising New Marker for Predicting Surgical Site Infection in Children With Ulcerative Colitis
Source: Ann Gastroenterol Surg. 2026 May 25:10.1002/ags3.70236. Online ahead of print. doi: 10.1002/ags3.70236 (PMC13394443; doi:10.1002/ags3.70236)
Supplement: Supplementary file 1 — Figure S1: Association between preoperative steroid exposure and LCR. There was no significant correlation between the total dose of preoperative steroids and the LCR values (p = 0.19). [file AGS3-9999-0-s002.pptx]

## Slide 1
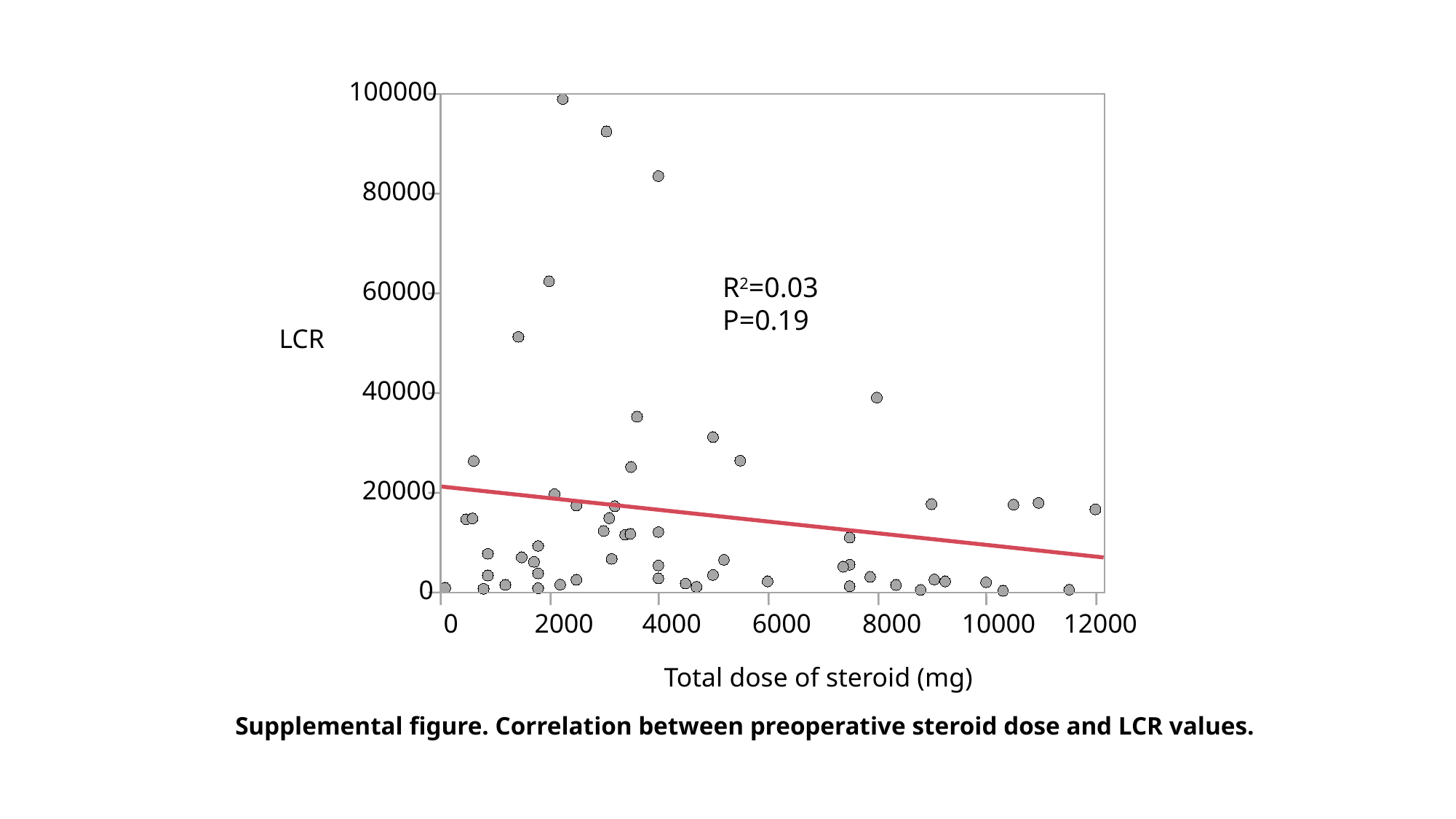

100000
80000
60000
LCR
40000
20000
0
0
2000
4000
6000
8000
10000
12000
Total dose of steroid (mg)
R2=0.03
P=0.19
Supplemental figure. Correlation between preoperative steroid dose and LCR values.
